# Comparative Study on the Efficacy of Intravitreal and Subconjunctival Simultaneous Injection of Conbercept Followed by Trabeculectomy Versus Intravitreal Injection of Conbercept Followed by Trabeculectomy Combined With 5‐Fluorouracil (5‐FU) in the Treatment of Neovascular Glaucoma (NVG)

**DOI:** 10.1155/joph/6946973

**Published:** 2026-10-27

**Authors:** Peiyu Xing, Wei Qu, Yong Zhang, Xiaojia Hu, Heman Xu, Bo Meng, Hao Shao, Shaowei Wang

**Affiliations:** ^1^ Department of Ophthalmology The 2nd Affiliated Hospital of Harbin Medical University Harbin China hrbmush.edu.cn; ^2^ Department of Ophthalmology China Medical University the Fourth People’s Hospital of Shenyang Shenyang China

**Keywords:** 5-fluorouracil, conbercept, neovascular glaucoma, neovascularization of the angle, neovascularization of the iris, trabeculectomy

## Abstract

**Purpose:**

To compare the efficacy of trabeculectomy preceded by simultaneous intravitreal and subconjunctival conbercept administration with that of trabeculectomy combined with 5‐fluorouracil (5‐FU) following intravitreal conbercept injection in patients with neovascular glaucoma (NVG).

**Methods:**

In this comparative study, 120 patients with NVG were enrolled and allocated to two treatment groups. Regression of neovascularization of the iris (NVI) and neovascularization of the angle (NVA) following conbercept administration was assessed. Additional outcome measures included intraocular pressure (IOP), visual acuity, filtering bleb morphology, use of antiglaucoma medications, surgical success rate, and postoperative complications before and after trabeculectomy.

**Results:**

On day 1 after conbercept injection, the group receiving simultaneous intravitreal and subconjunctival conbercept demonstrated significantly greater regression of NVI (*p* = 0.001) and NVA (*p* = 0.003) than the comparator group. Across follow‐up visits, significant reductions in IOP were observed within both groups (time effect: *F* = 352.42, *p* = 0.001). Improvement in visual acuity did not differ significantly between groups (*p* > 0.05). Significant between‐group differences in bleb height were observed at 1, 3, and 6 months after trabeculectomy (*p* = 0.008, *p* = 0.001, and *p* = 0.001, respectively). No significant difference in bleb extent was detected at any postoperative timepoint (*F* = 2.546, *p* = 0.080). Bleb vascularity differed significantly between groups throughout follow‐up (*F* = 3.297, *p* = 0.021). Rates of postoperative complications, antiglaucoma medication use before surgery and within 6 months after injection, and overall surgical success did not differ significantly between groups (all *p* > 0.05).

**Conclusion:**

In patients with NVG undergoing trabeculectomy, simultaneous intravitreal and subconjunctival administration of conbercept was associated with more rapid regression of anterior‐segment neovascularization and more favorable postoperative bleb characteristics. However, no significant between‐group differences were observed in 6‐month IOP control or surgical success. The potential benefits of the combined administration strategy appear to be mainly related to early neovascular regression and bleb morphology, and their significance for long‐term surgical outcomes remains to be determined.

## 1. Introduction

Neovascular glaucoma (NVG) is a severe secondary glaucoma that arises predominantly as a consequence of retinal ischemia. It is frequently refractory to conventional treatment because of progressive neovascularization of the iris (NVI) and iridocorneal angle, fibrovascular membrane formation, and markedly elevated intraocular pressure (IOP). Consequently, NVG poses a substantial threat to vision, is associated with a high risk of profound visual loss, and may ultimately result in irreversible blindness if not promptly and effectively managed [1].

Retinal vascular disorders that compromise retinal perfusion, including central retinal vein occlusion (CRVO), diabetic retinopathy (DR), and ocular ischemic syndrome, are major causes of NVG. Retinal ischemia and hypoxia stimulate the upregulation of vascular endothelial growth factor (VEGF), which plays a central role in the pathogenesis and progression of NVG [1–3].

Early diagnosis coupled with immediate and aggressive treatment is imperative to prevent complete vision loss in NVG patients [4, 5]. The primary therapeutic approach for NVG is panretinal photocoagulation (PRP), which improves the ischemic retina and results in shrinkage of abnormal blood vessels [3]. PRP plays a pivotal role in reducing the metabolic demand of the retina, consequently decreasing VEGF levels and neovascularization [6].

Short‐term treatment with NVG has shown efficacy in intravitreal administration of anti‐VEGF medications [6]. It induces abnormal vascular regression and helps to reduce intraoperative and postoperative complications [7, 8]. The clinical practice guidelines of the Japan Glaucoma Society highlight the potential benefits of preoperative intravitreal anti‐VEGF therapy in patients undergoing surgery for NVG, as this approach may reduce intraoperative and postoperative complications [9].

In cases of advanced NVG, filtration surgery may be necessary. However, excessive postoperative scarring in the conjunctiva and Tenon’s capsule at the sclerostomy site can lead to filtration failure, poor IOP control, and subsequent progression of glaucomatous disc cupping and visual field loss [10]. Fibroblasts in the Tenon’s capsule are crucial for ocular wound healing [11]. Although antimitotic medications such as mitomycin‐C (MMC) and 5‐fluorouracil (5‐FU) can reduce scarring after surgery and enhance surgical results, they can also lead to serious complications such as hypotony, blebitis, endophthalmitis, and wound leakage [12].

Previous studies have indicated elevated VEGF expression after glaucoma filtration surgery [13]. VEGF promotes the production of TGF‐b1 in the subconjunctival region, triggering the transformation of myofibroblasts and resulting in fibrosis at the site of the bleb [14]. Moreover, VEGF stimulates the growth of Tenon fibroblasts in a laboratory setting [15]. Inhibition of VEGF may enhance trabeculectomy outcomes by altering the postoperative fibrotic wound reaction [16].

Trabeculectomy aims to create a filtration channel through a scleral incision to drain the aqueous humor, reduce wound scarring, and establish functional follicles under the conjunctiva, ultimately lowering IOP [17]. Conbercept, a new anti‐VEGF drug from Chengdu Kanghong Biotech Co in Sichuan, China, at a concentration of 10 mg/mL in 0.2 mL per piece, has the potential to enhance trabeculectomy results by blocking VEGF and preventing scarring during glaucoma filtration surgery, suggesting that anti‐VEGF drugs have both direct and indirect effects on fibroblasts, suggesting the possibility of using them to enhance glaucoma filtering surgery [18]. To identify a safer and more effective adjunct for filtering surgery in patients with NVG, we compared the outcomes of Intravitreal and Subconjunctival Simultaneous Injection of Conbercept Followed by Trabeculectomy versus Intravitreal Injection of Conbercept Followed by Trabeculectomy Combined with 5‐FU.

## 2. Materials and Methods

### 2.1. Study Population

This comparative study was conducted at the Second Affiliated Hospital of Harbin Medical University and included 120 patients (120 eyes) diagnosed with NVG. Ethical approval was obtained from the Research Ethics Committee of the Second Affiliated Hospital of Harbin Medical University, Harbin, China, in accordance with the principles of the Declaration of Helsinki. All participants provided written informed consent after being fully informed about the study procedures, follow‐up schedule, and potential benefits and risks.

Participants were randomly assigned to one of the two treatment groups using a pre‐established randomization list of 120 numbers generated by a computerized random number generator (https://www.graphpad.com). Allocation concealment was ensured using sequentially numbered, sealed opaque envelopes prepared by an independent investigator who was not involved in patient enrollment, surgery, or postoperative assessment. Group assignment was revealed only after patient enrollment. Because of the nature of the surgical interventions, masking of the surgeon and patients was not feasible. However, postoperative examinations, bleb grading, and data collection were performed by trained ophthalmologists who were masked to treatment allocation.

A total of 120 patients with NVG were randomly assigned to two groups (*n* = 60 per group). The experimental group received intravitreal and subconjunctival conbercept injections combined with trabeculectomy, whereas the control group received intravitreal conbercept injection followed by trabeculectomy with intraoperative 5‐FU application. Both groups underwent PRP.

Inclusion criteria were: (1) unilateral NVG secondary to CRVO, DR, or ocular ischemic syndrome; (2) age 40–78 years; (3) elevated IOP (> 21 mmHg); (4) use of anti‐glaucoma medications; and (5) presence of iris or angle neovascularization.

Exclusion criteria included: (1) other ocular comorbidities such as keratoconus, lens dislocation, uveitis, retinal disorders, congenital ocular abnormalities, or ocular trauma; (2) known allergy to conbercept (3) uncontrolled systemic diseases (e.g., diabetes mellitus or hypertension); increasing surgical risk; (4) pregnancy or lactation; (5) prior intravitreal anti‐VEGF treatment or previous glaucoma surgery; (6) inability to adequately assess lens status due to media opacity; and (7) inability to comply with follow‐up requirements.

### 2.2. Study Outcomes

Comprehensive demographic and clinical data, including medical and ocular history, best‐corrected visual acuity (BCVA), and IOP, were collected for all participants. Standardized case report forms were used to record data at 1 day, 1 week, 1 month, 3 months, and 6 months postoperatively. Follow‐up assessments included evaluation of BCVA, NVI and neovascularization of the angle (NVA), IOP measurement, documentation of antiglaucoma medication use, detailed assessment of filtering bleb morphology, and monitoring for postoperative complications.

NVI and NVA in the study eye were evaluated using the NVI and NVA grading systems, respectively, on a scale ranging from 0 to 4 [19]. Filtering bleb morphology was carefully assessed by masked examiners using the Indiana Bleb Appearance Grading Scale (IBAGS). Bleb characteristics were systematically evaluated, including height (H0–H3), extent (E0–E3), vascularity (V0–V4), and leakage (S0–S2) [20].

Each examination and grading was performed once at each follow‐up visit by a single trained observer. To ensure consistency, a standardized photographic reference set was used. To minimize potential bias, assessment of bleb characteristics was completed prior to IOP measurement.

At 6 months, the primary outcome was surgical success. Complete success was defined as IOP ≤ 21 mmHg with a reduction of ≥ 30% from baseline, absence of hypotony maculopathy, no requirement for additional glaucoma surgery, and preservation of at least light perception (LP) vision. Eyes requiring antiglaucoma medications to meet these criteria were classified as qualified success. Failure was defined as IOP > 21 mmHg or a reduction of < 30% from baseline. Complete failure was defined as loss of LP vision or the need for additional glaucoma surgery.

Secondary outcomes included BCVA, NVI, NVA, IOP, number of glaucoma medications, bleb morphology, and postoperative complications.

### 2.3. Study Procedures

#### 2.3.1. Intravitreal and Subconjunctival Injections

All conbercept injections and filtration surgeries were performed by the same experienced surgeon following a standardized protocol. Intravitreal injections were carried out in an operating room under strict aseptic conditions. Patients received topical levofloxacin eye drops four times daily for 3 days preoperatively. Before injection, oxybuprocaine hydrochloride eye drops were administered three times for topical anesthesia, followed by instillation of 5% povidone‐iodine into the conjunctival sac, which was rinsed after 30 s. Conbercept (0.5 mg/0.05 mL) was injected via a 30‐gauge needle 4 mm posterior to the superotemporal or inferotemporal limbus through the pars plana. In the experimental group, an additional subconjunctival injection of conbercept (1.0 mg/0.1 mL) was administered.

#### 2.3.2. Trabeculectomy

Three days after conbercept injection, all patients underwent trabeculectomy under topical and peribulbar anesthesia using 0.5% lidocaine eye drops. After eyelid speculum placement, a fornix‐based conjunctival flap was created, followed by the formation of a half‐thickness scleral flap (4 × 4 mm). The scleral flap was dissected to expose the trabecular meshwork. In the control group, a sponge soaked in 5‐FU was applied beneath the scleral flap for a short duration and then irrigated with balanced saline solution, whereas no 5‐FU was used in the experimental group. Anterior chamber paracentesis was performed to lower IOP. A trabecular block measuring approximately 2 × 1 mm was excised, followed by peripheral iridectomy. The scleral flap and conjunctiva were closed with 10‐0 nylon sutures.

Postoperatively, patients received tobramycin and dexamethasone eye drops four times daily, along with cycloplegic eye drops three times daily for 2 weeks, with a gradual taper over the subsequent 2 weeks.

#### 2.3.3. PRP

The procedure was performed 2–3 times using a power range of 200–400 mW, an exposure time of 0.2–0.3 s, and a spot size of 200–300 μm, with a total of 1500–2000 burns applied. In each session, the ophthalmologist ensured complete coverage of the peripheral retina, maintaining procedural consistency across all treatments.

### 2.4. Statistical Analysis

SPSS software (version 20.0; SPSS Inc., Chicago, IL, USA) was used for all statistical analyses. The normality of continuous variables was assessed using the Shapiro–Wilk test. Normally distributed data were analyzed using parametric tests, whereas non‐normally distributed data were analyzed using appropriate nonparametric methods. The chi‐square test was used for categorical variables, nonparametric tests for rank variables, and the independent‐samples *t*‐test for continuous variables. Repeated‐measures analysis of variance (ANOVA) was used to evaluate IOP, as measurements were obtained from the same subjects at multiple postoperative time points, allowing assessment of within‐subject temporal changes. Generalized linear mixed models (GLMM) were applied for ordinal outcomes (e.g., bleb grading) to account for repeated measurements and potential deviations from normal distribution. A two‐sided *p* value < 0.05 was considered statistically significant.

## 3. Results

### 3.1. Participants’ Characteristics

Table 1 summarizes the baseline characteristics of all patients. No statistically significant differences were observed between the two groups.

**TABLE 1:** Demographic and baseline characteristics of patients.

Parameters	Experimental group	Control group	t/c2	*p*
No. of patients	60	60		
Mean age ± SD	56.97 ± 11.00	59.40 ± 8.94	0.940	0.351
IOP at presentation	47.41 ± 13.15	48.02 ± 13.66	0.176	0.861
Gender (%)				
Male	40 (66.7%)	40 (66.7%)	0.000	1.000
Female	20 (33.3%)	20 (33.3%)		
Retinal ischemic disease				
DR	38 (63.3%)	42 (70.0%)	0.300	0.584
CRVO	22 (36.7%)	18 (30.0%)		
NVA/NVI grading systems				
NVI				
1	0 (0%)	4 (6.7%)	5.343	0.346
2	20 (33.3%)	14 (23.3%)		
3	28 (46.7%)	26 (43.35)		
4	12 (20.0%)	16 (26.7%)		
NVA				
1	16 (26.7%)	16 (26.7%)	4.467	0.728
2	42 (70.0%)	32 (53.3%)		
3	2 (3.3%)	12 (20%)		
Lens (%)				
Phakic	52 (86.7%)	48 (80%)	0.480	0.488
Pseudophakia	8 (13.3%)	12 (20%)		
Presenting vision				
FC	6 (10%)	12 (20%)	2.747	0.432
HM	18 (30%)	24 (40%)		
LP	20 (33.3%)	12 (20%)		
VA	16 (26.7%)	12 (20%)		
PRP				
Preoperative	36 (60%)	32 (53.3%)	0.271	0.602
Postoperative	24 (40%)	28 (46.7%)		

*Note:* IOP, intraocular pressure; FC, count finger; PRP, panretinal photocoagulation; NVA, neovascularization of the angle; NVI, neovascularization of the iris.

Abbreviations: CRVO, central retinal vein occlusion; DR, diabetic retinopathy; HM, hand movement; LP, light perception.

### 3.2. IOP

Table 2 presents IOP changes over time. Repeated‐measures analysis demonstrated a significant temporal reduction in IOP across the postoperative follow‐up period (*F* = 352.43, *p* < 0.001). However, no statistically significant between‐group difference in IOP reduction was observed during follow‐up.

**TABLE 2:** Intraocular pressure (IOP) at 1 day, 1 week, 1, 3, and 6 months after filtration surgery in both groups.

Timen	Experimental group	Control group
Baseline	47.41 ± 13.15	48.02 ± 13.66
Day = 1	18.12 ± 3.42	18.89 ± 3.50
Day = 1 week	17.70 ± 3.50	18.22 ± 4.60
Day = 1 month	17.55 ± 2.49	17.56 ± 3.10
Day = 3 months	17.15 ± 1.86	17.56 ± 2.79
Day = 6 months	16.29 ± 1.73	17.90 ± 2.51
F	352.43	
*p*	< 0.001	

*Note:* ANOVA, repetitive measure analysis of variance. *p* < 0.05, significant difference.

### 3.3. Postoperative Visual Acuity (VA)

For statistical analysis, CF, HM, and LP were converted into logMAR equivalents based on previously published methods (CF = 2.1, HM = 2.4, LP = 2.7) [21]. Figure 1 shows a gradual improvement in VA in both groups over time. However, no significant differences in VA improvement were observed between the two groups at any follow‐up time point (*p* > 0.05).

**FIGURE 1:**
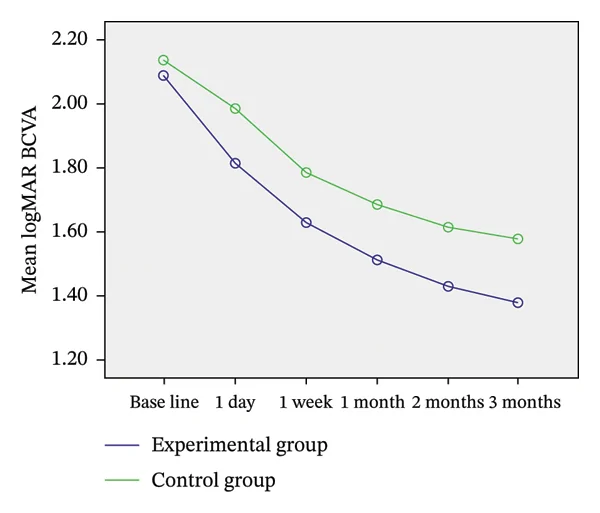
VA changes in both groups before and after filtration surgery 1 day, 1 week, 1, 3, and 6 months (GEE). *Note:* *p* > 0.05, no significant difference. Abbreviation: GEE: generalized estimating equations.

### 3.4. NVA/NVI

Table 3 shows neovascularization data. A significantly higher regression rate of both NVI and NVA was observed in the experimental group compared with the control group on postoperative day 1 following conbercept injection (NVI, *p* = 0.001; NVA, *p* = 0.003).

**TABLE 3:** NVI/NVA on pre‐injection, post‐injection at 1 day in both groups.

Time	Experimental group	Control group	Mann–Whitney U	*p*
NVI Pre‐operation				
1	0 (0%)	4 (6.7%)	395	0.346
2	20 (33.3%)	14 (23.3%)		
3	28 (46.7%)	26 (43.35)		
4	12 (20.0%)	16 (26.7%)		
NVI Post‐injection 1 day				
0	22 (36.7%)	8 (13.3%)	246	0.001
1	30 (50.0%)	24 (40.0%)		
2	8 (13.3%)	28 (46.7%)		
NVA Pre‐operation				
1	16 (26.7%)	16 (26.7%)	428	0.728
2	42 (70.0%)	32 (53.3%)		
3	2 (3.3%)	12 (20%)		
NVA Post‐injection 1 day				
1	34 (56.7%)	12 (20%)		0.003
2	26 (43.3%)	36 (60.0%)		
3	0 (0%)	12 (20%)		

*Note:* *p* < 0.05 significant difference.

### 3.5. Filtering Bleb Morphology

Tables 4–6 summarize bleb morphology based on IBAGS. No significant differences in bleb height (H) were observed between groups at day 1 (*p* = 0.068) and 1 week (*p* = 0.073). However, significant differences were observed at 1 month (*p* = 0.008), 3 months (*p* = 0.001), and 6 months (*p* = 0.001).

**TABLE 4:** Type of conjunctival bleb height (H) at 1 day, 1 week, 1, 3 and 6 months after filtration in both groups (GLMM).

Time	Experimental group			Control group			*p*
grading systems	1	2	3	1	2	3	
Day = 1	16 (26.7%)	40 (66.7%)	4 (6.7%)	28 (46.7%)	32 (53.3%)	0 (0%)	0.068
Day = 1 week	24 (40.0%)	36 (60.0%)	0 (0%)	38 (63.3%)	22 (36.7%)	0 (0%)	0.073
Day = 1 month	28 (46.7%)	32 (53.3%)	0 (0%)	48 (80.0%)	12 (20.0%)	0 (0%)	0.008
Day = 3 months	34 (56.7%)	26 (43.3%)	0 (0%)	56 (93.3%)	4 (6.7%)	0 (0%)	0.001
Day = 6 months	38 (63.3%)	22 (36.7%)	0 (0%)	58 (96.7%)	2 (3.3%)	0 (0%)	0.001

*Note:* *p* < 0.05 significant difference.

Abbreviation: GLMM, generalized linear mixed models.

**TABLE 5:** Type of conjunctival bleb extent (E) at 1 day, 1 week, 1, 3 and 6 months after filtration in both groups (GLMM).

Time	Experimental group			Control group		
grading systems	1	2	3	1	2	3
Day = 1	20 (33.3%)	40 (66.7%)	0 (0%)	14 (23.3%)	42 (70.0%)	4 (6.7%)
Day = 1 week	28 (46.7%)	32 (53.3%)	0 (0%)	18 (30.0%)	42 (70.0%)	0 (0%)
Day = 1 month	38 (63.3%)	22 (36.7%)	0 (0%)	28 (46.7%)	32 (53.3%)	0 (0%)
Day = 3 months	48 (80.0%)	12 (20.0%)	0 (0%)	30 (50.0%)	30 (50.0%)	0 (0%)
Day = 6 months	44 (73.3%)	16 (26.7%)	0 (0%)	26 (43.3%)	34 (56.7%)	0 (0%)

*Note:* Group main effect: *F* = 2.546, *p* = 0.080. Time main effect: *F* = 1.996, *p* = 0.047.

Abbreviation: GLMM, generalized linear mixed models.

**TABLE 6:** Type of conjunctival bleb vascularity (V) at 1 day, 1 week, 1, 3 and 6 months after filtration in both groups (GLMM).

Time	Experimental group				Control group			
Grading systems	0	1	2	3	0	1	2	3
Day = 1	0 (0%)	4 (6.7%)	34 (90.0%)	2 (3.3%)	0 (0%)	4 (6.7%)	50 (83.3%)	6 (10.0%)
Day = 7	0 (0%)	18 (30.0%)	42 (70.0%)	0 (0%)	0 (0%)	32 (53.3%)	28 (46.7%)	0 (0%)
Day = 30	0 (0%)	36 (60.0%)	24 (40.0%)	0 (0%)	0 (0%)	56 (93.3%)	4 (6.7%)	0 (0%)
Day = 90	14 (23.3%)	46 (76.7%)	0 (0%)	0 (0%)	24 (40%)	36 (60.0%)	0 (0%)	0 (0%)
Day = 180	36 (60.0%)	24 (40.0%)	0 (0%)	0 (0%)	42 (70.0%)	18 (30.0%)	0 (0%)	0 (0%)

*Note:* Group main effect: *F* = 3.297, *p* = 0.021. Time main effect: *F* = 4.973, *p* = 0.031.

No significant between‐group difference in bleb extent (E) was found at any time point (*F* = 2.546, *p* = 0.080), although significant within‐group temporal changes were observed (*F* = 1.996, *p* = 0.047).

Bleb vascularity (V) differed significantly between groups at all postoperative time points (*F* = 3.297, *p* = 0.021), with significant temporal changes within groups as well (*F* = 4.973, *p* = 0.031).

### 3.6. Complications

Table 7 summarizes postoperative complications. No intraoperative complications occurred. Mild complications such as hyphema, corneal epithelial defects, shallow anterior chamber, and choroidal detachment were slightly more frequent in the control group; however, differences between groups were not statistically significant (*p* = 1.000). No cases of transient ciliary body detachment, endophthalmitis, or hypotony were observed in either group.

**TABLE 7:** Complications in both groups.

Complications	Experimental group	Control group	*p*
Hyphema	0	1	1.000
Corneal epithelial stripping	0	1	1.000
Shallow anterior chamber	1	2	1.000
Endophthalmitis	0	0	
Choroidal detachment	0	1	1.000
Ciliary body detachment	0	0	
Hypotony (IOP < 5 mm Hg)	0	0	

*Note:* *p* > 0.05, no significant difference.

### 3.7. Antiglaucoma Medications

Table 8 shows the number of antiglaucoma medications at baseline and 6 months postoperatively. No significant differences were observed between groups before or after surgery (*p* > 0.05).

**TABLE 8:** No. of medications for pre‐operation and post‐injection 6 months in both groups.

Time	Experimental group	Control group	*p*
Pre‐operation	3.07 ± 0.41	3.00 ± 0.45	0.735
Post‐injection 6 months	0.18 ± 0.37	0.39 ± 0.64	0.210

*Note:* *p* > 0.05, no significant difference.

### 3.8. Surgical Success

Table 9 presents 6‐month surgical outcomes. No significant difference in overall surgical success was observed between groups (*p* = 0.948), defined as IOP < 21 mmHg with ≥ 30% reduction from baseline. Cases requiring additional glaucoma surgery were classified as complete failure. No patient lost LP vision during follow‐up.

**TABLE 9:** Treatment outcomes at 6 months (*n* = 60 per group).

Outcome	Experimental group (*n* = 60)	Control group (*n* = 60)	*p*
Complete success	46 (76.7%)	44 (73.3%)	0.948
Qualified success	10 (16.7%)	10 (16.7%)	
Failure	2 (3.3%)	4 (6.7%)	
Complete failure	2 (3.3%)	2 (3.3%)	

*Note:* IOP, intraocular pressure. *p* > 0.05, no significant difference.

## 4. Discussion

NVG poses a significant challenge in its management due to its refractory nature arising from retinal ischemia and hypoxia. Common treatment modalities include intravitreal anti‐VEGF injections combined with filtering surgery or drainage procedures for aqueous humor [1]. However, the success of glaucoma filtration surgery is often impeded by excessive scarring, which is a major obstacle to achieving effective IOP control and preventing disease progression [22]. The development of fibrous tissue at the site of surgery, especially in the area where the conjunctiva, Tenon’s capsule, and episclera meet, plays a crucial role in the creation of a successful bleb after surgery [22]. Bleb failure resulting from heightened scarring leads to disease progression and inadequate IOP control [23]. Modern glaucoma surgeons often use antifibrotic medications such as 5‐FU and MMC to regulate wound healing, improve bleb longevity, and control IOP [24].

While antifibrotic agents effectively target fibroblast activity, particularly their transformation and proliferation, their nonselective cell death mechanism can lead to severe postoperative complications, including hypotony, avascular blebs, late bleb leaks, and endophthalmitis [25, 26]. Despite the predominant focus on fibroblast activities in interventions, it is crucial to recognize the role of angiogenesis in the wound‐healing process [27]. Angiogenesis plays a crucial role in wound healing by facilitating the transportation of different mediators and regulators to the core of the healing process [28]. Excessive vascularization of the conjunctiva, accompanied by fibroblast migration due to cytokine release, contributes to bleb failure and triggers the wound healing response, culminating in fibrosis [28]. Fibroblasts in Tenon’s capsule, which are responsible for creating collagen and elastin, have a crucial impact on the development of scars in the eye following filtration surgery [29]. Both human and rabbit Tenon fibroblasts express VEGF receptors at the mRNA and protein levels, and studies have demonstrated that VEGF stimulates Tenon fibroblast proliferation in vitro, whereas anti‐VEGF treatments at different concentrations inhibit fibroblast proliferation [20].

We chose to administer subconjunctival injection simultaneously with intravitreal conbercept for several reasons. Previous studies have demonstrated upregulated VEGF gene expression and increased levels of other cytokines in postoperative bleb tissues in rat models 48 h after glaucoma filtration surgery [30]. Animal experiments have shown that the time after subconjunctival injection of fusion protein in rabbit eyes can reach 7 days, allowing the drug to continue its effects even after filtration surgery is performed 2–3 days later [31]. Second, the economic cost and acceptance by patients pose challenges when using anti‐VEGF in filtering surgery. The use of 5‐FU in these procedures can cause widespread and indiscriminate cell destruction, leading to serious complications after surgery, such as low IOP, thin bloodless blebs, delayed bleb ruptures, and inflammation within the eye [32].

Both treatment groups demonstrated a significant reduction in IOP from baseline following trabeculectomy. However, no statistically significant difference in IOP reduction was observed between the two groups during the 6‐month follow‐up. The significant temporal effect observed in the repeated‐measures analysis indicates that IOP decreased substantially after surgery in both groups, whereas the adjunctive anti‐VEGF strategies did not produce a demonstrable difference in IOP control within the current follow‐up period. A previous study also reported a significant decrease in IOP in patients receiving bevacizumab, 5‐FU, or placebo, with no significant differences in final mean IOP among the three groups [33]. Although the present study had a 6‐month follow‐up period, which is generally considered sufficient to detect clinically meaningful changes in IOP, further studies with larger sample sizes and longer follow‐up durations are warranted to validate and strengthen these findings.

Regarding VA improvement, no significant difference was observed between the two groups, although both demonstrated a gradual and sustained improvement following surgery. These findings are consistent with a previous study involving 74 patients with NVG who underwent trabeculectomy followed by conbercept injection, which reported a significant improvement in BCVA [34]. Notably, we observed that eyes presenting with initial LP achieved progressive improvement in VA, reaching logMAR 1.6, 1.5, and 1.4 over the follow‐up period in the experimental group.

Our study demonstrated a significant difference in the regression of NV between the two groups after the initial anti‐VEGF injection, with a faster regression observed in the experimental group compared with the control group. This effect may be attributed to the additional subconjunctival injection, which, while maintaining a constant intravitreal dose, could enhance drug availability across multiple ocular tissues, thereby contributing to more rapid NV regression. Experimental studies have further supported this mechanism, showing that subconjunctival administration of aflibercept and bevacizumab can reach target tissues such as the retinal pigment epithelium (RPE)/choroid complex and retina, effectively suppressing VEGF activity [31–35]. A clinical study injecting bevacizumab solely into the subconjunctiva of NVG patients also reported rapid regression of iris or angle neovascularization accompanied by a reduction in IOP [36]. Our study aligns with these findings and supports the hypothesis that simultaneous intravitreal and subconjunctival injection of anti‐VEGF is a novel approach for the rapid reduction of neovascularization.

Although no significant differences were observed in IOP reduction or surgical success rate at 6 months between the two groups, the experimental group demonstrated earlier regression of NVI/NVA and more favorable bleb morphology. This apparent discrepancy may be explained by the fact that trabeculectomy itself is highly effective in rapidly reducing IOP in the short to mid‐term, potentially masking differences between adjunctive treatment strategies within a 6‐month observation window.

Importantly, early suppression of neovascularization and reduced bleb vascularity may still have important clinical implications for long‐term outcomes. Excessive postoperative angiogenesis and fibrovascular proliferation are key contributors to late bleb scarring and surgical failure. Therefore, the observed improvements in early vascular regression and bleb characteristics may translate into benefits for long‐term bleb survival, which could not be fully captured within the current follow‐up period.

Accordingly, the clinical impact of the present findings should be interpreted in the context of short‐ to mid‐term outcomes, and longer follow‐up is required to determine whether these early advantages lead to sustained differences in surgical success.

Scar inhibition, specifically the morphology of blebs, was the focus of our study. In the past 10 years, many research projects have investigated the effectiveness of bevacizumab/conbercept as a treatment for preventing scarring following trabeculectomy in both animal models (rabbits) and patients with glaucoma, generally yielding favorable outcomes, and bevacizumab subconjunctival injections in an animal model of glaucoma filtration surgery resulted in blebs that were more durable, larger, elevated, and had reduced scarring when compared to the control group [37]. Another animal study demonstrated that subconjunctival injection of conbercept after trabeculectomy significantly reduced the inflammatory response during incision healing and decreased neovascularization and fibroblasts, ultimately inhibiting scar formation [38].

A clinical trial with 51 patients divided into bevacizumab, 5‐FU, and placebo groups showed notable variation in central bleb size and vascularity as assessed by bleb structure analysis [33]. In a separate study, 36 eyes that underwent surgery for open‐angle glaucoma were randomly divided into groups that received either subconjunctival injection of conbercept or 5‐FU. The conbercept group showed reduced vascularity values compared with the 5‐FU group at different time points after injection [39].

However, it is important to note that neither the animal models nor the patient populations in the aforementioned studies specifically involved NVG. In addition, several studies have demonstrated that aqueous humor VEGF levels are elevated in patients with glaucoma prior to filtering surgery, based on evidence from animal models, in vitro experiments, and human clinical studies [30]. There are also studies indicating that VEGF levels in the aqueous humor of glaucoma patients increase after filtration surgery [40, 41], which distinguishes NVG from other types of glaucoma.

In this study, we assessed changes in filtering bleb morphology using the IBAGS system and observed improvements in bleb height, extent, and vascularity at different follow‐up time points in the control group. A previous meta‐analysis demonstrated that trabeculectomy combined with intravitreal anti‐VEGF injection achieves a significantly higher success rate in NVG patients compared with trabeculectomy alone, suggesting improved bleb morphology and function. We hypothesize that intravitreal administration of anti‐VEGF agents allows adequate diffusion within intraocular tissues and fluids, thereby contributing to modulation of postoperative wound healing and inhibition of excessive bleb scarring [35]. Variations in bleb height (H), bleb extent (E), and bleb vascularity (V) at different follow‐up times between the two groups could be due to the more immediate and enduring effects of anti‐VEGF medications administered via subconjunctival injection [31]. This is supported by studies showing a longer duration of action for bevacizumab after subconjunctival injection than after intravitreal injection [35].

Although our study highlights the potential advantages of combined intravitreal and subconjunctival anti‐VEGF injection in patients with NVG undergoing trabeculectomy, several limitations should be acknowledged. First, the relatively modest sample size and single‐center design may limit the generalizability of our findings. Second, the 6‐month follow‐up period may be insufficient to evaluate long‐term filtration surgery success, bleb survival, and late postoperative complications. Third, masking of surgeons and participants was not feasible because of the nature of the surgical interventions, although postoperative outcome assessments were performed by masked examiners. Therefore, larger multicenter prospective studies with longer follow‐up are warranted to validate these findings and determine whether the observed early advantages in neovascular regression and bleb morphology translate into improved long‐term surgical outcomes.

## 5. Conclusions

In patients with NVG undergoing trabeculectomy, simultaneous intravitreal and subconjunctival administration of conbercept was associated with more rapid regression of NVI/NVA and more favorable postoperative bleb characteristics than intravitreal conbercept followed by trabeculectomy combined with 5‐FU. However, no significant between‐group differences were observed in 6‐month IOP control, medication use, postoperative complications, or the primary endpoint of surgical success. Therefore, the potential clinical benefits of combined intravitreal and subconjunctival conbercept should be interpreted cautiously, particularly with respect to long‐term filtration success. Larger, multicenter prospective studies with longer follow‐up are warranted to determine whether the observed early improvements in neovascular regression and bleb morphology translate into sustained clinical benefits.

## Funding

This work was supported by the Beijing Bethune Charitable Foundation (grant number: BJ2020IIT002).

## Disclosure

The funding had no role in the study design, data collection and analysis, decision to publish, or preparation of the manuscript.

## Conflicts of Interest

The authors declare no conflicts of interest.

## Data Availability

The data used to support the findings of this study are available from the corresponding author upon request.
